# Resveratrol inhibits the progression of premature senescence partially by regulating v-rel avian reticuloendotheliosis viral oncogene homolog A (RELA) and sirtuin 1 (SIRT1)

**DOI:** 10.1080/0886022X.2022.2029488

**Published:** 2022-02-15

**Authors:** Shuangjun He, Meng Zhou, Hongming Zheng, Yaowei Wang, Shuhua Wu, Yuan Gao, Jianhong Chen

**Affiliations:** a Department of Orthopedic Surgery, Affiliated Danyang Hospital of Nantong University, The People’s Hospital of Danyang, Danyang, China; b Department of Orthopedic Surgery, Tengzhou Central People’s Hospital, Tengzhou, China; c Department of Forensic Science, Medical School of Soochow University, Suzhou, China

**Keywords:** Premature senescence, resveratrol, RELA, sirtuin 1, apoptosis

## Abstract

**Objective:**

To explore the effect of resveratrol in premature senescence and reveal its anti-premature senescence mechanisms through network pharmacology.

**Methods:**

In this study, the H_2_O_2_-induced bone marrow mesenchymal stem cells (BMMSCs) premature senescence model is applied. Cell counting kit-8 assay, β-galactosidase staining and flow cytometry are conducted to detect the proliferation, senescence and apoptosis of BMMSCs. Bioinformatics analyses are used to screen and validate molecular targets of resveratrol acting on premature senescence. Dual-luciferase reporter assay is conducted to verify the interaction between v-rel avian reticuloendotheliosis viral oncogene homolog A (RELA) and sirtuin 1 (SIRT1). RT-qPCR and western blot are adopted to detect mRNA and protein levels of RELA, SIRT1, senescence-related genes and apoptosis-related genes.

**Results:**

First, we proved that resveratrol alleviated the H_2_O_2_-induced senescence of BMMSCs. Then, bioinformatics analysis revealed that RELA was the downstream target of resveratrol and SIRT1 was the downstream target of RELA, respectively, involved in premature aging. RELA/SIRT1 may be the potential target of resveratrol for premature senescence. Notably, rescue experiments indicated that resveratrol inhibited premature senescence partially through targeting regulation RELA/SIRT1.

**Conclusion:**

In our study, we confirm the functional role of the resveratrol-RELA- SIRT1 axis in the progression of premature senescence, which provides a latent target for premature senescence treatment.

## Introduction

Cellular senescence is a process in which cell proliferation, differentiation and physiological function gradually decline with the passage of time in the process of life activities. It is a permanent state of cell cycle arrest that can be triggered by a variety of stressors, including DNA damage, telomere shortening, and oxidative stress [1,2]. According to different activation signals, cellular senescence is classified into two categories: replicative senescence and premature senescence. Replicative senescence, as Hayflick and Moorhead found in normal mammalian cells, is characterized by a limited replication potential that limits its lifespan to a certain number of divisions [3]. Currently, as is known to all, it is induced by telomere shortening triggered signals [4]. Premature senescence refers to senescence induced in young cells through several other mechanisms, such as activation of certain oncogenes, inactivation or mitotic stimulation of tumor suppressor genes, DNA damage factors, and oxidative stress [5]. Cells that experience premature senescence are morphologically indistinguishable from replicative senescence cells and exhibit many features attributed to replicative senescence, such as the increased activity of age-associated β-galactase (SA-β-gal) and increased expression of p53 and p21 [6,7]. In recent years, despite some progress in the study of senescence, the biological mechanism of senescence has remained poorly understood. Both physiological senescence and pathological senescence are based on the overall senescence of the organism’s cells. Therefore, to elucidate the mechanism of the organism’s senescence must start from studying the mechanism of cell senescence.

Since currently few ideal treatments and medicines reverse premature senescence or slow the progress of the disease, much more attention has been attached to Chinese herbs and extracts [8]. Recently, scientific efforts have focused on the potential role of traditional herbal extracts as an alternative and complementary medicine for premature senescence [9]. Resveratrol (trans-3,4′,5-trihydroxystibene) is a nontoxic, naturally occurring polyphenolic compound found in many plants, such as grapes, red wine, and mulberries. Recent clinical studies have shown that resveratrol is well tolerated and relatively safe in humans [10,11]. In addition, resveratrol has been confirmed to have a variety of biological functions, including anti-oxidant, anti-inflammatory, anti-senescence, heart protection, nerve protection, etc. [12,13]. Interestingly, studies in cancer and animal models have shown that resveratrol inhibits tumor cell activity and induces premature senescence [14,15]. Seabra et al. have shown that resveratrol reduces H_2_O_2_-induced fibroblast senescence. Besides, research by Ali et al. [16] has shown that resveratrol inhibit adipocyte differentiation and senescence of human bone marrow stromal stem cells (BMMSCs). However, the underlying mechanism by which resveratrol inhibits cell senescence remains unclear.

In this study, bioinformatics and molecular biology methods were used to explore the mechanism of resveratrol regulating the progression of premature senescence.

## Methods

### Cell culture and treatment

BMMSCs were obtained from American Type Culture Collection (ATCC, Manassas, VA). After all the cells were resuscitated, they were cultured in RPMI-1640 medium containing 10% FBS and 100 units/mL penicillin, 100 g/mL streptomycin, 2 mmol/L glutamine in a humidified environment and the medium were changed every other day. For the induction of premature senescence, sub-confluent BMMSCs were exposed to 100 μM H_2_O_2_ for 2 h [17]. Then the cells were washed twice with phosphate buffer to remove H_2_O_2_ and re-cultured in a fresh complete medium.

### Cell counting kit-8 (CCK-8) assay

The number of viable cells of BMMSCs was detected by the CCK-8 (Beyotime, Beijing, China). In short, the cells adjusted to the appropriate concentration were inoculated on 96-well plates and treated accordingly. Then, each well was added with CCK-8 solution and incubated for 2 h in the dark. Finally, the optical density at 450 nm was measured.

### β-galactosidase staining

First, untreated BMMSCs and treated BMMSCs were both fixed with 0.5% glutaraldehyde. After being washed with PBS, the cells were stained in X-gal solution overnight at 37 °C. Subsequently, all stained cells were observed with an inverted bright field microscope (Olympus, Tokyo, Japan), and approximately 500 cells were counted in random fields to determine the percentage of blue-stained β-galactosidase positive cells.

### Flow cytometry

First, BMMSCs were suspended in ice PBS and then centrifuged for 5 min. Second, after washing, the samples were incubated in buffer solution with FITC-labeled Annexin-V (Carlsbad, CA) for 15 min at room temperature in dark of light. Third, propidium iodide (PI, 640932, Biolegend, San Diego, CA) was added and stored on ice in the dark of light. Finally, the cell apoptosis was immediately tested on the flow cytometer (FACScan, BD Biosciences, Beijing, China). CELL Quest 3.0 software (BD Biosciences, Beijing, China) was used to analyze data.

### Prediction of putative targets of resveratrol and RELA

To identify the potential targets of resveratrol, Swiss Target Prediction (http://www.swisstargetprediction.ch/), Comparative Toxicogenomics Database (http://ctdbase.org/), Protein Analysis Through Evolutionary Relationships (PANTHER, http://pantherdb.org/), Metascape (http://metascape.org/), and Gene Ontology (http://geneontology.org) were used. Protein–RNA Interface Database (PRIDB, http://bindr.gdcb.iastate.edu/) was used to predict and screen the putative targets of RELA.

### Chemical structure analysis of resveratrol

The chemistry structural information of resveratrol was searched, collected, and confirmed with PubChem (https://pubchem.ncbi.nlm.nih.gov/).

### Three-dimensional modeling of RELA

Briefly, the amino acid sequence of RELA was first obtained from the NCBI BLAST search program. Then, the amino acid sequence was input into SWISSMODEL to construct the 3D models of RELA by the Protein Modeling Server.

### Docking studies

Docking studies were performed by MGL tools (version 1.5.6) and Autodock 4.2. First, MGL tools were used to convert the file type and Pymol was used to preprocess the protein model file. Then Autodock was conducted to calculate the possibility of binding between resveratrol molecules and RELA protein molecules. All configurations of the receptor and ligand complexes were analyzed by Software company Accelrys, with each color bar representing a binding pattern of the chemical molecules.

### Quantitative real-time PCR

TRIpure reagent (Invitrogen, Carlsbad, CA) was used to extract the total RNA from BMMSCs and PrimeScript RT kit (TaKaRa, Tokyo, Japan) was used for reverse transcription. After the sample was prepared, the expression level was detected by SYBR green, and β-actin was controlled as an internal parameter. The primer sequences for this experiment were shown below: RELA, GAATGGCTCGTCTGTAGTG and antisense, TGGTATCTGTGCTCCTCTC; SIRT1 sense, GCAGATTAGTAGGCGGCTTG and antisense TCTGGCATGTCCCACTATCA; β-actin sense, TCACCAACTGGGACGACATG and antisense, GTCACCGGAGTCCATCACGAT.

### Western blot

According to the manufacture’s instruction, the proteins were extracted and their concentrations were measured. Subsequently, the prepared protein was separated by polyacrylamide–SDS gels and then transferred onto PVDF membranes (Roche, Basel, Switzerland). Blocked with 5% nonfat dry milk for 2.5 h, the PVDF membranes were subjected to incubation with primary antibodies against RELA, SIRT1, Bax, Bcl-2, p53, p21 and β-actin (1:1000, Proteintech Group Inc., Wuhan, China) at 4 °C overnight. On the following day, protein samples were incubated with the secondary antibody at 37 °C for 45 min and the intensity of protein expression was detected by ECL chemiluminescence.

### Cell transfection

pc-NC, pc-RELA, si-NC, si-SIRT1, and pc-SIRT1 were constructed by Ribobio Corporation (Guangzhou, China). When the confluence rate of BMMSCs reached 70–80%, the transfection was conducted by using Lipofectamine 2000 (Invitrogen, Carlsbad, CA), and plasmid dosage per transfection was 100 ng.

### Dual-luciferase reporter assay

First, wt-SIRT1 and mut-SIRT1 were inserted into pmirGLO reporter vectors (Promega, Madison, WI), respectively. Then, BMMSCs were plated onto 24-well plates the day before transfection and co-transfected with luciferase reporter vectors and pc-RELA or negative control (pc-NC). Finally, luciferase activities were determined with the Dual-Luciferase Reporter System (Promega, Madison, WI).

### Statistical analysis

All the data were analyzed by Statistical Package for Social Sciences19.0 (SPSS, Chicago, IL). One-way ANOVA followed by Dunnett’s multiple comparisons was applied to assess the differences between the groups. The results were presented (mean ± SD). Differences were considered significant at *p* < 0.05.

## Results

### Resveratrol alleviates H_2_O_2_-induced senescence of BMMSCs

**Figure 1.**
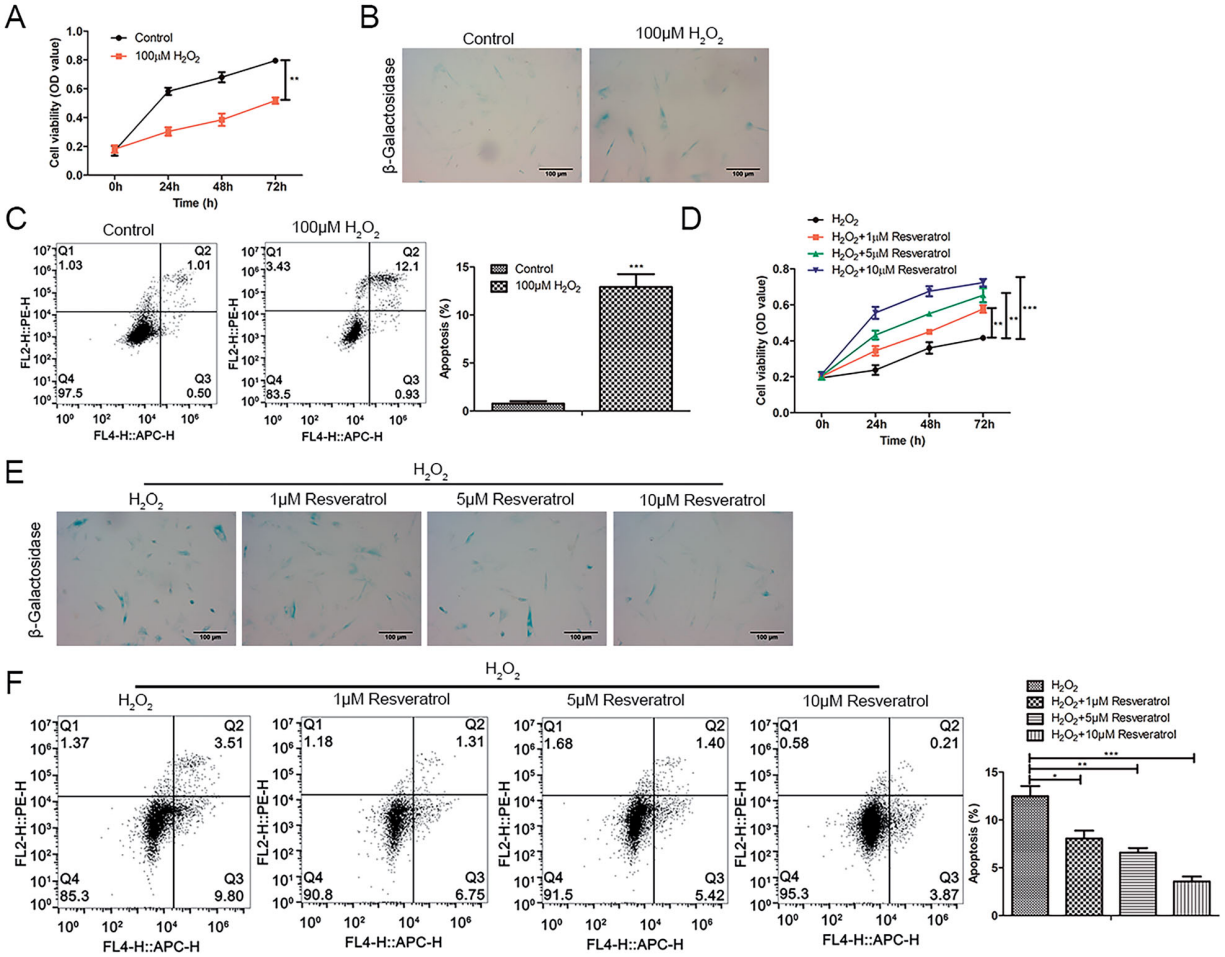
Resveratrol promotes H_2_O_2_-induced proliferation activity and inhibits senescence and apoptosis of BMMSCs. Proliferation, senescence and apoptosis of BMMSCs treated with H_2_O_2_ were detected by (A) CCK-8 assay, (B) β-galactosidase staining assay and (C) flow cytometry assay, respectively. After treatment with different concentrations of resveratrol (1 µM, 5 µM, and 10 µM) for 24 h, the proliferation, senescence and apoptosis of H_2_O_2_-induced BMMSCs were detected by (D) CCK-8 assay, (E) β-galactosidase staining assay, and (F) flow cytometry assay. *p < 0.05, **p < 0.01, ***p < 0.001 versus the control group.

As presented in Figure 1(A), H_2_O_2_ produced cell cytotoxicity and reduced BMMSCs activity in a time-dependent pattern as comparisons to the control group. The β-galactosidase staining results showed that the proportion of β-galactosidase positive cells stained in blue was significantly increased (Figure 1(B)). Meanwhile, the results of flow cytometry showed that H_2_O_2_ induced apoptosis (Figure 1(C)). To investigate the role of resveratrol in premature senescence, H_2_O_2_-induced BMMSCs were treated with resveratrol (1, 5 and 10 µM). In the CCK-8 assay, we found that resveratrol reduced H_2_O_2_-induced premature senescence in a time-dependent and concentration-dependent manner (Figure 1(D)). Consistently, in β-galactosidase staining and flow cytometry, the addition of resveratrol significantly decreased premature senescence positive cells and apoptotic cells in a concentration-dependent manner (Figure 1(E,F)).

### RELA is the target of resveratrol in premature senescence

**Figure 2.**
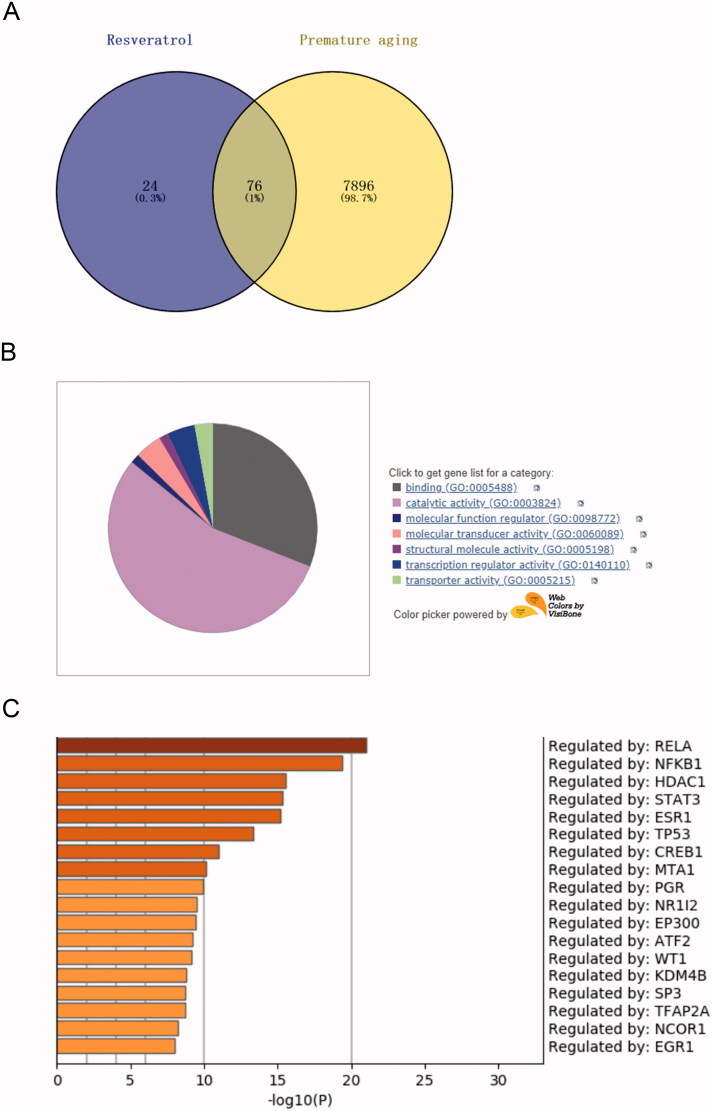
RELA is the target of resveratrol in premature senescence. (A) Swiss Target Prediction and Comparative Toxicogenomics Database were used to identify the potential targets of resveratrol; (B) the biological function of the selected target genes was analyzed by PANTHER and gene ontology; (C) Metascape and gene ontology were used to select genes with higher expression from the selected target genes with transcriptional activity.

**Figure 3.**
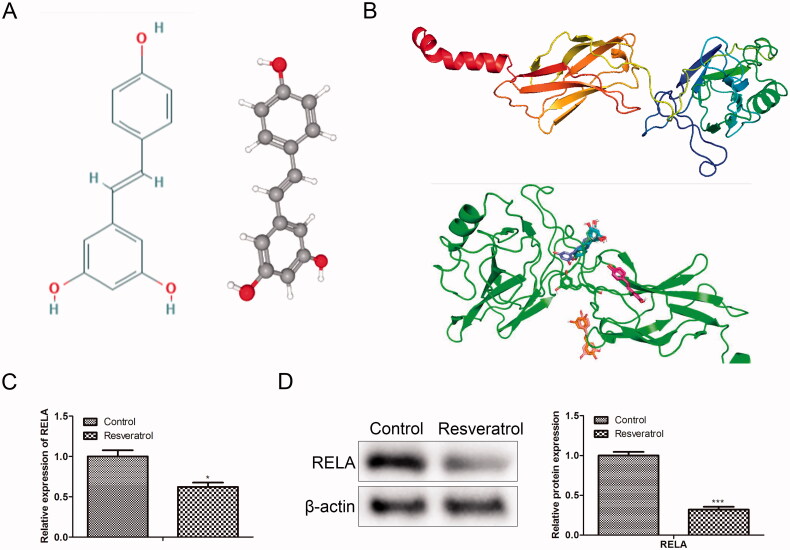
Simulate and verify the combination of resveratrol and RELA. (A) Chemical structure of resveratrol; (B) the 3D structure model of RELA was conducted by SWISSMODEL and Autodock was used to calculate the possibility of binding between resveratrol molecules and RELA protein molecules; (C) the mRNA levels of RELA were detected by RT-qPCR; (D) the protein levels of RELA were detected by western blot. *p < 0.05, ***p < 0.001 versus the control group.

To clarify the regulatory mechanism of resveratrol in premature senescence, we conducted a bioinformatics analysis. From the Comparative Toxicogenomics Database, 7972 targets related to premature senescence were obtained. Through Swiss Target Prediction analysis, 100 targets of resveratrol were obtained. Crosswise, a total of 76 related targets of resveratrol in premature senescence were identified (Figure 2(A)). Further, to screen out an effective target from the 76 identified targets, GO analysis was performed using databases PANTHER and Metascape. In detail, from the PANTHER database, we observed that the identified 76 target genes were primarily associated with binding, catalytic activity, molecular function regulator, molecular transducer activity, structural molecule activity, transcription regulator activity and transporter activity, among which the transcriptional activity was the most important function (Figure 2(B)). After that, Metascape was applied to select the target gene with high activity from the genes with transcriptional activity. As presented in Figure 2(C), RELA was an effective target gene of resveratrol involved in premature senescence. To confirm this inference, we next simulated and verified the binding of resveratrol and RELA. The chemical structure of resveratrol and the 3D structure of RELA was shown in Figure 3(A,B), respectively. Specifically, as shown in Figure 3(B), Autodock algorithm revealed the top 10 binding ways of resveratrol and RELA. The green bars represented protein molecules, and each colored bar represented a way that chemical molecules bound together. Besides, RT-qPCR and western blot provided further evidence that resveratrol bound to RELA and promoted its degradation (Figure 3(C)).

### Overexpression of RELA weakens the effect of resveratrol in the treatment of premature senescence

**Figure 4.**
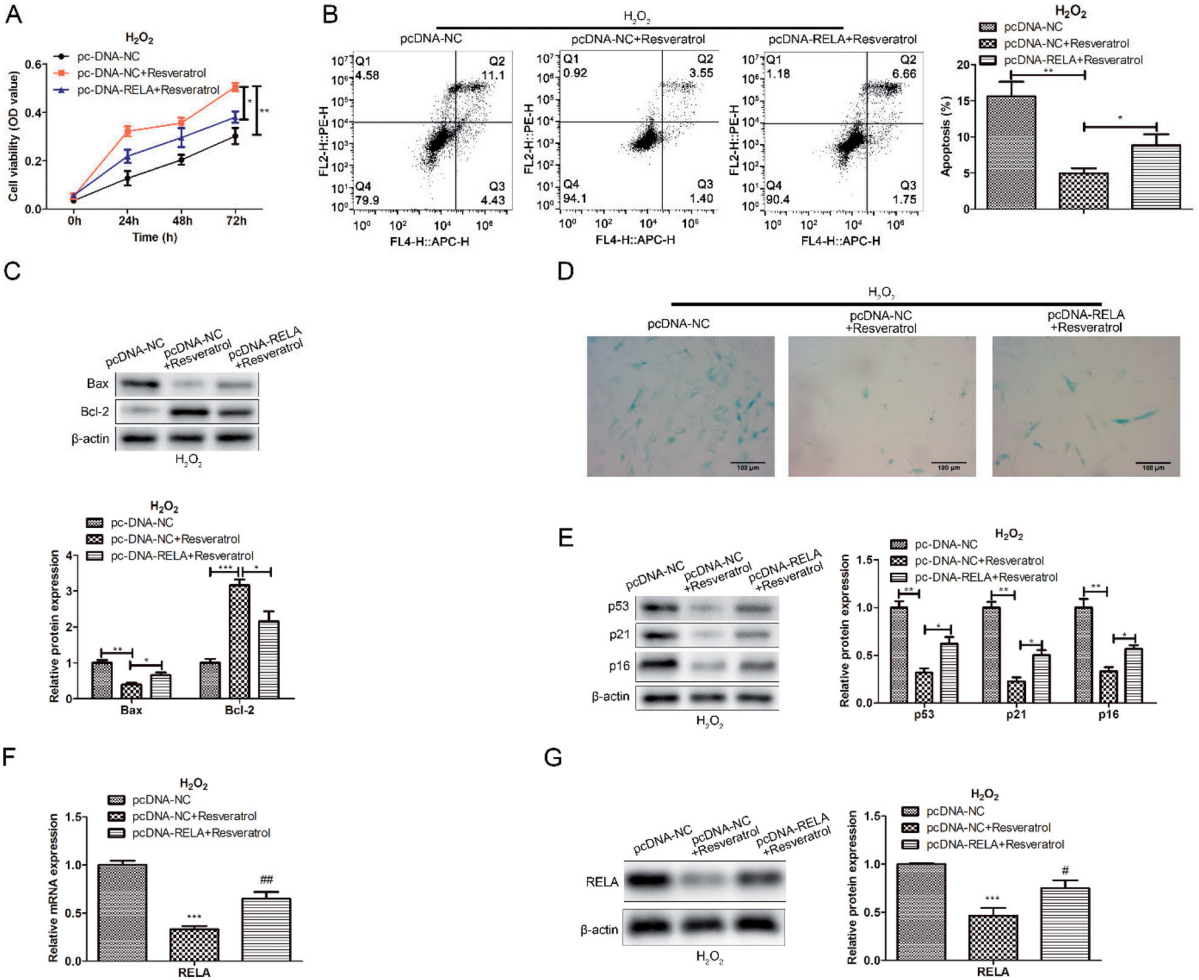
Overexpression of RELA weakens the effect of resveratrol in the treatment of premature senescence. (A) CCK-8 and (B) flow cytometry were used to detect BMMSCs proliferation activity and apoptosis rate induced by H_2_O_2_ in groups pc-NC, pc-NC + resveratrol and pc-RELA + resveratrol; (C) the expression of apoptosis-related proteins (Bax and Bcl-2) was detected by western blot; (D) β-galactosidase staining assay was performed to determine the senescence of BMMSCs; (E) the expression of senescence-related proteins (p53 p21 and p16) was detected by western blot; (F) the mRNA expression of RELA was detected by qRT-PCR; (G) the expression of RELA was detected by western blot. *p < 0.05, **p < 0.01, ***p < 0.001 versus another group.

To further explore the relationship between resveratrol and RELA in the progression of premature senescence, we first constructed the RELA overexpression vector. Next, BMMSCs were co-transfected with pc-NC or pc-NC + resveratrol or pc-RELA + resveratrol to detect the effects on proliferation and apoptosis. As exhibited in Figure 4(A), compared with transfection pc-NC + resveratrol, transfection pc-RELA + resveratrol enhanced the H_2_O_2_-induced cytotoxicity. In the apoptosis assay, overexpression of RELA inhibited the protective effect of resveratrol on BMMSCs to a certain extent (Figure 4(B)). Consistently, western blot results showed an increased expression in Bax protein and a decreased expression in Bcl-2 protein after overexpression of RELA, indicating that RELA promoted apoptosis (Figure 4(C)). In the β-galactosidase staining assay, after co-transfection with RELA, the proportion of β-galactosidase-positive cells in blue was significantly increased, along with an increase in p53 and p21 proteins associated with premature senescence (Figure 4(D,E)). These results indicated that overexpression of RELA weakened the therapeutic effect of resveratrol on premature senescence.

### SIRT1 is the target of RELA in premature senescence

**Figure 5.**
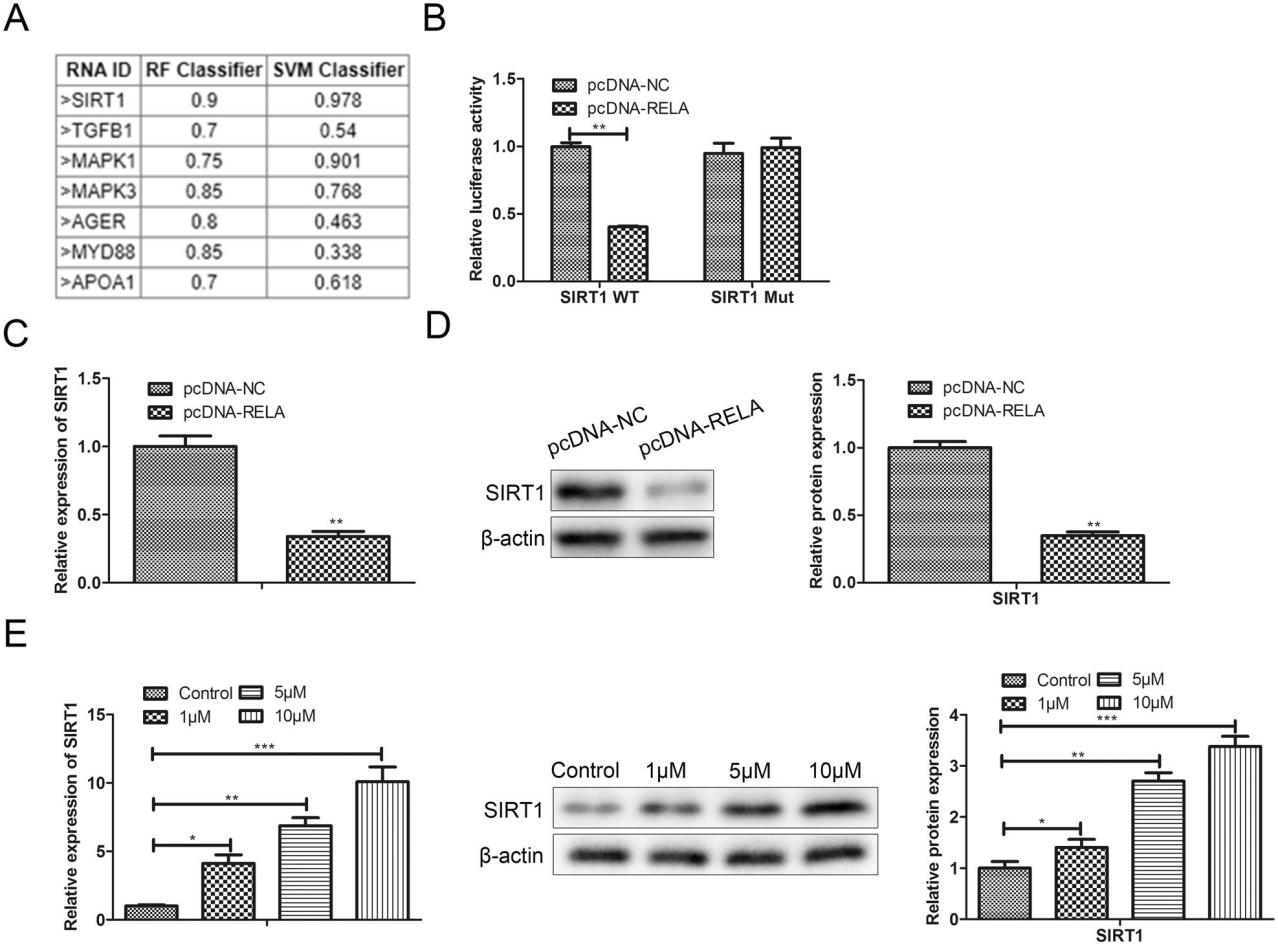
SIRT1 is the target of RELA in premature senescence. (A) PRIDB was used to predict and screen the putative targets of RELA; (B) dual-luciferase reporter assay is conducted to verify the interaction between RELA and SIRT1; (C) after overexpression of RELA, the mRNA expression of SIRT1 were detected by qRT-PCR; (D) after overexpression of RELA, the protein expression of SIRT1 were detected by western blot; (E) the mRNA and protein expression of SIRT1 after treatment with different concentrations of resveratrol (1 µM, 5 µM, and 10 µM) for 24 h were evaluated by qRT-PCR and western blot. *p < 0.05, **p < 0.01, ***p < 0.001 versus another group.

To clarify the regulatory mechanism of RELA in premature senescence, we evaluated the RNA binding degree of RELA protein and premature senescence-related genes by PRIDB, and found that RELA protein had a high possibility of RNA binding with SIRT1 (Figure 5(A)). Therefore, we speculated that SIRT1 might be the target gene of RELA involved in the regulation of premature senescence. To verify this prediction, we conducted two types of luciferase reporter gene vectors (SIRT1-wt and SIRT1-mut) and carried out a luciferase reporter assay. As depicted in Figure 5(B), the relative luciferase activity of SIRT1-3′-UTR-wt was remarkably reduced in pc-RELA transfected BMMSCs compared with pc-NC transfected cells. However, when this binding site was mutated, over-expression of RELA did not affect luciferase activity. Besides, both RT-qPCR and western blot results illustrated that over-expression of RELA inhibited SIRT1 expression at both transcriptional and translational levels (Figure 5(C)). Meanwhile, we observed that resveratrol increased the mRNA level and protein level of SIRT1 in a dose-dependent manner (Figure 5(D)).

### Silencing SIRT1 weakens the effect of resveratrol in the treatment of premature senescence

**Figure 6.**
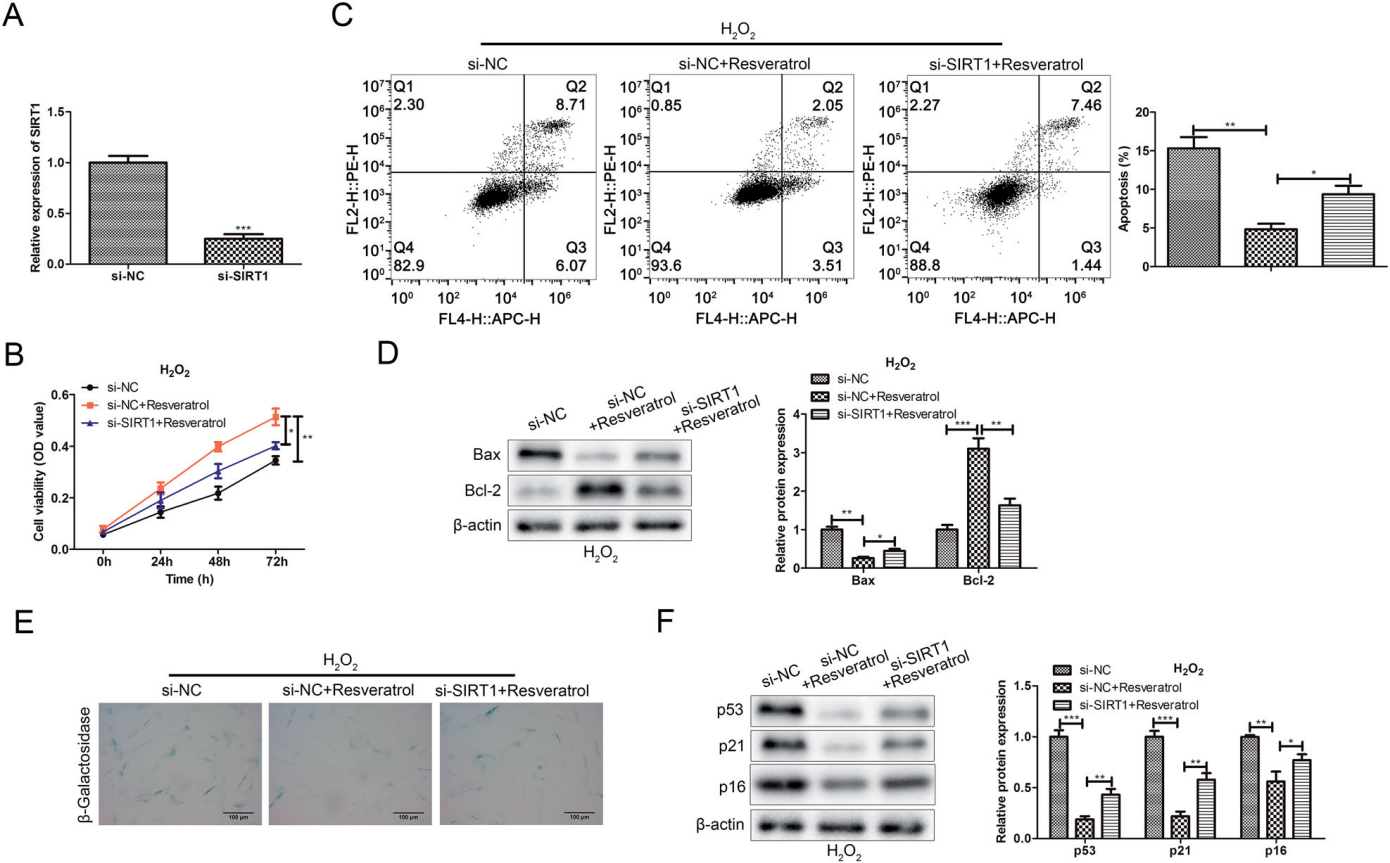
Silencing SIRT1 weakens the effect of resveratrol in the treatment of premature senescence. (A) Efficiency of SIRT1 silence was tested. (B) CCK-8 and (C) flow cytometry were used to detect BMMSCs proliferation activity and apoptosis rate induced by H_2_O_2_ in groups si-NC, si-NC + resveratrol and si-RELA + resveratrol; (D) the expression of apoptosis-related proteins (Bax and Bcl-2) was detected by western blot; (E) β-galactosidase staining assay was performed to determine the senescence of BMMSCs; (F) the expression of senescence-related proteins (p53, p21 and p16) was detected by western blot. *p < 0.05, **p < 0.01, ***p < 0.001 versus another group.

To further explore the relationship between resveratrol and SIRT1 in the progression of premature senescence, we silenced the expression of SIRT1 and detected its effects on cell proliferation, apoptosis and premature senescence. In the CCK-8 assay, compared with the si-NC ± resveratrol group, the si-SIRT1+ resveratrol group significantly attenuated H_2_O_2_-induced cytotoxicity (Figure 6(A)). In flow cytometry assay, the Annexin V positive cells in the si-SIRT1 + resveratrol group were significantly increased in comparison to the si-NC + resveratrol group (Figure 6(B)). In western blot assay, silencing SIRT1 sharply increased the protein expression of Bax and conversely decreased the expression of Bcl-2 in H_2_O_2_-induced BMMSCs (Figure 6(C)). In the β-galactosidase staining and western blot assay, after the transfection with si-SIRT1, we observed that the proportion of β-galactosidase-positive cells in blue was significantly increased, along with an increase in p53 and p21 proteins associated with premature senescence (Figure 6(D,E)).

### Resveratrol inhibits premature senescence partially through targeted regulation RELA/SIRT1

**Figure 7.**
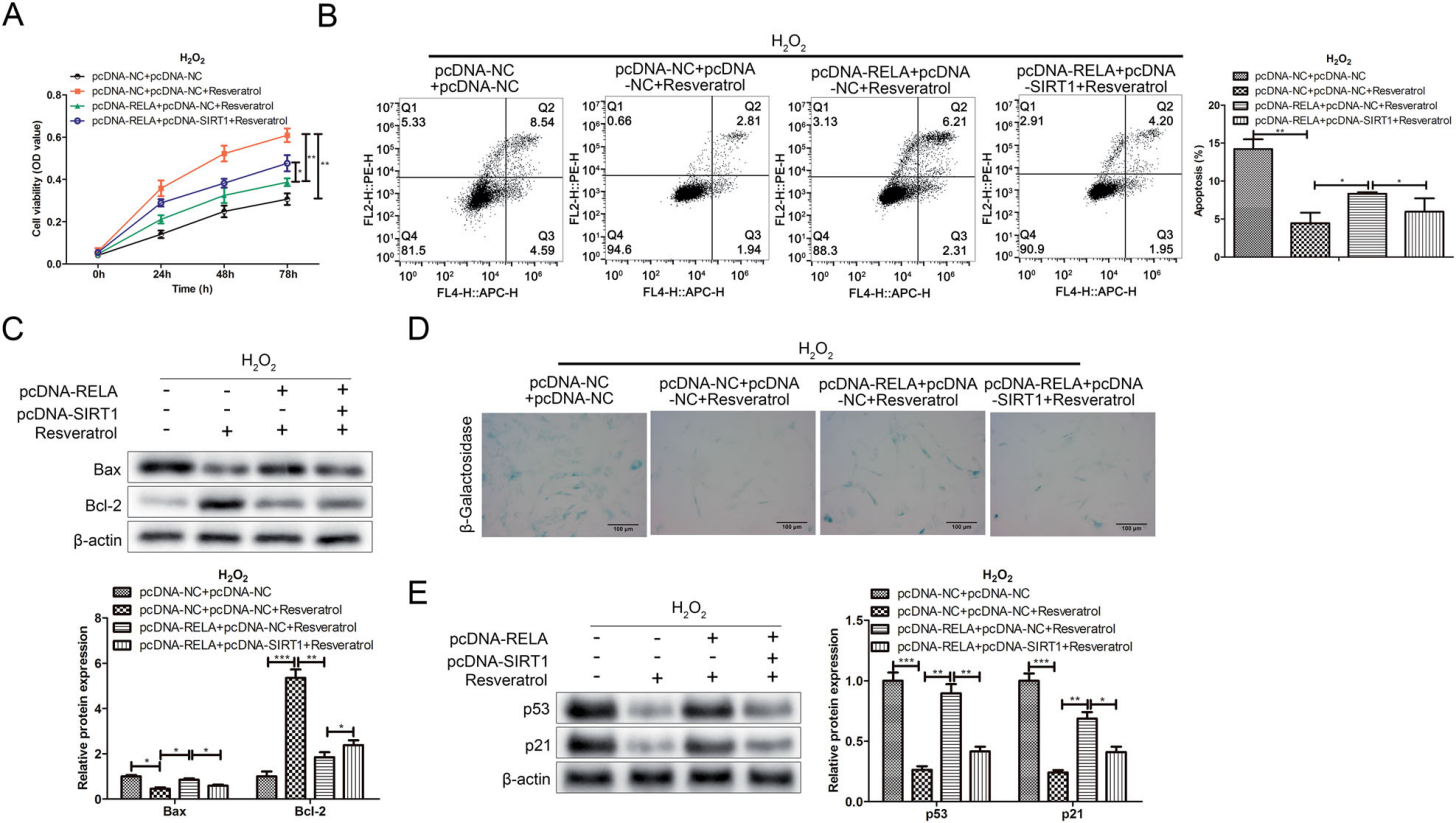
Resveratrol inhibits premature senescence through targeted regulation RELA/SIRT1. (A) CCK-8 and (B) flow cytometry were used to detect BMMSCs proliferation activity and apoptosis rate induced by H_2_O_2_ in group pc-NC + pc-NC, pc-NC + pc-NC + resveratrol, pc-RELA + pc-NC + resveratrol and pc-RELA + pc-SIRT1+ resveratrol; (C) the expression of apoptosis-related proteins (Bax and Bcl-2) was detected by western blot; (D) β-galactosidase staining assay was performed to determine the senescence of BMMSCs; (E) the expression of senescence-related proteins (p53, p21 and p16) was detected by western blot. *p < 0.05, **p < 0.01, ***p < 0.001 versus another group.

To further study whether resveratrol executed its function in premature senescence *via* RELA/SIRT1 axis, rescue assays were performed. As shown in the CCK-8 results in Figure 7(A), resveratrol increased H_2_O_2_-induced BMMSCs activity compared with the pc-DNA-NC + pc-DNA + NC group. However, after the co-transfection with pc-RELA, the cell proliferation activity was significantly reduced in the pc-DNA-RELA + pc-DNA-NC + resveratrol group as comparison to the pc-DNA-NC + pc-DNA + NC + resveratrol group. Notably, the co-transfection with pc-SIRT1 offset the inhibitory effect of pc-RELA on resveratrol promoting BMMSCs proliferation compared with the pc-DNA-RELA + pc-DNA-NC + resveratrol group. Further, in related experiments to verify apoptosis (flow cytometry and western blot), the results both proved that resveratrol influenced cell apoptosis by regulating RELA/SIRT1 (Figure 7(B,C)). Besides, we observed that the transfection with pc-RELA significantly weakened the effect of resveratrol in the treatment of premature senescence, accompanied by an increase in the proportion of β-galactosidase-positive cells in blue and the expression of premature senescence-related proteins. On the contrary, co-transfection pc-SIRT1 significantly reversed the promoting effect of pc-RELA on premature senescence (Figure 7(D,E)).

## Discussion

Oxidative stress is one of the most important mechanisms of cell senescence. Therefore, stress-based cell senescence models have been widely used to understand the senescence process and identify potential regulatory factors of cell senescence. Among the most commonly used inducers for stress premature senescence, H_2_O_2_ is a classic substance that induces cell senescence [18]. Based on the above background, we used H_2_O_2_ as an inducer to establish a premature senescence model of BMMSCs and then carried out an in-depth exploration of the anti-premature senescence mechanism of resveratrol. Consistently, in our study, we found that H_2_O_2_ induced premature senescence of BMMSCs. However, the addition of resveratrol partially slowed the H_2_O_2_-induced premature senescence of BMMSCs in a concentration-dependent manner.

Bioinformatics, a technology that uses computational techniques to organize and integrate experimental data, has been well applied in many researches [19]. Swiss Target Prediction is a webserver for target prediction of bioactive small molecules. Using a combination of 2D and 3D similarity measures, it compares the query molecule to a library of 280 000 compounds active on more than 2000 targets of 5 different organisms [20]. Comparative Toxicogenomics Database is a major public resource for literature-based, manually curated associations between chemicals, gene products, phenotypes, diseases, and environmental exposures. It provides information on chemical/gene, protein interactions, chemical diseases, and genetic diseases [21]. The PANTHER database contains comprehensive information on the evolution and function of protein-coding genes from 104 complete sequenced genomes. The software tool allows users to classify new protein sequences and analyze lists of genes obtained from large-scale genomic experiments [22]. Metascape integrates Go, KEGG, Uniprot and Drugbank databases to enable not only pathway enrichment and biological process annotation, but also gene-related protein network analysis and drug analysis [23]. As a comprehensive database of protein-RNA interfaces extracted from complexes in the Protein Data Bank (PDB), PRIDB provides researchers with atomic and residue-level information about protein–RNA complexes and the structure of their interfaces, facilitating the analysis of protein–RNA interactions [24]. Gene ontology defines and describes the functions of genes and proteins. It contains three functional information about the biological processes involved in genes, the composition of cells, and the molecular functions performed, and it organizes functional concepts with different concepts into the structure of DAG [25]. Molecular docking is a theoretical simulation method to study the interaction between molecules (such as ligand and receptor) and predict their binding patterns and affinity, which is used to design drugs based on the characteristics of the receptor and the interaction mode between the receptor and drug molecules [26]. In this study, the above bioinformatics tools were used to predict and verify that RELA was the downstream target of resveratrol and SIRT1 was the downstream target of RELA, respectively, involved in premature aging. Therefore, we conducted an *in vitro* study to further validate the role of resveratrol in inhibiting premature senescence by targeting the RELA/SIRT1 axis.

Nuclear factor kappa-B (NF-κB) protein was first discovered by David Baltimore, and this protein family can selectively bind to κ-light chain enhancers of B cells to regulate the expression of many genes. It is known for its important role in immune and inflammatory diseases [27]. NF-kB is composed of multiple subunits, but RELA has received the most attention and is considered to be a key member of the typical NF-κB pathway [28]. DiDonato et al. have shown that RELA has a potential role in the occurrence and development of cancer by regulating the expression of genes related to cell proliferation, migration, invasion, angiogenesis, and drug resistance to radiation or chemotherapy [29]. Treiber et al. have reported that RELA is required for fibrous formation in mice with chronic pancreatitis and that inhibition of RELA significantly reduced liver fibrosis [30]. In addition, RELA inhibition has been confirmed to prevent tendon adhesion by regulating inflammation, cell proliferation, and apoptosis [31]. However, it remained unclear whether RELA was involved in cell aging and what function it played. In the presented study, bioinformatics data showed that RELA was a downstream target gene of resveratrol involved in premature aging. Further, quantitative data provided further evidence that resveratrol bound to RELA and promoted its degradation. Resveratrol activates Sirtuin-1 (Sirt1) inhibiting RELA acetylation and promoting inhibitor protein-κBα (IkBα) degradation in the immune response and cardiovascular disease [32,33]. However, the mechanism by which resveratrol promotes RELA mRNA degradation remains unclear in this study. More study needs to be done. Besides, functional research data revealed that overexpression of RELA weakened the effect of resveratrol in the treatment of premature senescence. Taken together, RELA was an important target of resveratrol involved in premature aging.

Sirtuins, a class of proteins that retain both histone deacetylase and mononuclear transferase activity, are involved in biological processes such as aging, transcriptional regulation, apoptosis, stress resistance, and energy efficiency and alertness under calorie restriction [34,35]. In mammals, seven members of the sirtuin family (SIRT1–7) have been identified, located in different cellular structures such as nucleus (SIRT1, 2, 6, 7), cytoplasm (SIRT1 and SIRT2), and mitochondria (SIRT3–5) [36,37]. Among them, SIRT1 is the most commonly studied in recent years. SIRT1 deletion results in impaired gametophyte development, heart and retinal abnormalities, genomic instability, smaller body size, and low survival rate in mice [38,39]. Resveratrol, with the property of reducing oxidative stress-mediated renal injury in the animal model of nephropathy, can inhibit PTH induced apoptosis and restore bcl-2 expression as a sirtuin 1 agonist [40,41].

In BM-MSCs, SIRT1 delays senescence and promotes long-term growth by prolonging cell passage. Without SIRT1, early passage BMMSCs lose their self-renewal ability, and the expression of cell cycle inhibitors increases, thus accelerating cell senescence [42]. Consistently, here we reported the effect of knockdown expression of SIRT1 on the function of BMSCs induced by H_2_O_2_. Evidence showed that siRNA-mediated down-regulation of SIRT1 reduced BMMSCs proliferation activity, promoted senescence and apoptosis, and ultimately weakened the effect of resveratrol in the treatment of premature aging. More notably, rescue experiments confirmed that resveratrol inhibited cell senescence by targeting the RELA/SIRT1 axis [43,44]. This will offer a new direction for drug targets and systems drug designs for the treatment of premature aging. Still, additional work is needed to determine the upstream and downstream molecules of this pathway.

## Conclusion

In our study, a novel mechanism of anti-premature senescence by resveratrol was investigated. The study concluded that resveratrol can alleviate the senescence of the H2O2-induced BMMSCs premature senescence model. Mechanistically, we proved that resveratrol might exert its anti-premature senescence function partially through RELA/SIRT1.

## Data Availability

The raw data supporting the conclusions of this manuscript will be made available by the authors, without undue reservation, to any qualified researcher.
